# Social Capital and Mental Wellbeing of Older People Migrating along with Adult Children in Shenzhen, China

**DOI:** 10.3390/ijerph20196857

**Published:** 2023-09-28

**Authors:** Julia Juan Wang, Daniel W. L. Lai, Wenqing Yu

**Affiliations:** 1 Elderly Healthcare College, Shenzhen Polytechnic University, Shenzhen 518055, China; wangjuan@szpt.edu.cn; 2 Faculty of Social Sciences, Hong Kong Baptist University, Hong Kong, China; 3 Department of Social Work, Hong Kong Baptist University, Hong Kong, China; 21481482@life.hkbu.edu.hk

**Keywords:** older adults migrating along, social capital, mental wellbeing

## Abstract

The phenomenon of ‘older people migrating along’ (OPMA) with adult children is a unique outcome of social changes that have occurred in China. These individuals generally experience different challenges and needs associated with mental wellbeing. However, there is limited research on the relationship between the social capital and mental health of OPMA in China. This study aims to examine the mental health status of OPMA and the effects of bonding social capital and bridging social capital on their mental wellbeing in China by conducting a quantitative research survey among 399 OPMA participants. We found that bonding social capital correlated to only one indicator of mental wellbeing, subjective happiness. Bridging social capital had significant relationships with four mental health indicators, namely, the 12-item General Health Questionnaire (GHQ-12), Geriatric Depression Scale (GDS), subjective happiness, and life satisfaction. Through strengthening bridging social capital, these older adults can benefit from more opportunities for participation in formal or informal organizations in their communities and improve their mental wellbeing.

## 1. Introduction

With urbanization, economic reform, and social welfare reform in China, about 7.74 million older people have migrated to urban centers, where their adult children reside and work, to care for their grandchildren [1]. These older migrants are known as ‘older people migrating along’ (OPMA) with adult children (in Chinese: sui qian lao ren) or ‘older drifting group’ (lao piao zu) among researchers in Mainland China. The number of OPMA with adult children is likely to continue to rise, with China’s government aiming for urbanization to cover 60% of the population [2].

OPMA’s motivation to migrate is generally not to earn a living, but to help to care for their grandchildren. They are generally of retirement age, ranging from 50 years old to over 70 years old. They tend to live in cities for an indefinite length of time, probably without changing their ‘hukou’ registration, which is a form of residency record tied to one’s entitlement to social security and health benefit coverage based on one’s legal right of abode in their hometown.

While OPMA provide unpaid care for their grandchildren, they tend to experience challenges and needs associated with mental wellbeing, such as a higher level of depression than local older people, lower levels of quality of life (QoL), and negative feelings of rootlessness, loneliness, and depression [3,4]. Mental health has become one of the biggest challenges confronting these older migrants migrating along with their adult children [4].

Among the various key social determinants of mental health of older people, the role of social capital has gained more attention [5]. Social capital is found to be positively associated with the promotion of mental health and the prevention of mental illness for older adult populations and the Chinese population [6]. Currently, the government in China has also implemented some policies to enhance older adults’ mental health, such as setting up mental health rehabilitation centers in local hospitals and providing community education courses related to mental health knowledge in local communities [7]. These activities, implemented in local communities in Western countries, have shown positive effects on residents’ mental health literacy and enhancement of social capital of the users [8]. However, there is limited research on the relationship between the social capital and mental health of OPMA in China. This study aimed to examine the relationship between the social capital and mental health of OPMA based on a sample in one of China’s major cities. Mental wellbeing, reflecting the equilibrium between an individual and their environment in a broad sense [9], is a state of wellbeing in which an individual realizes their own abilities, copes with life stressors, works productively and fruitfully, and can make contributions to their community [10]. A recent literature review by the authors [4] has shown that older migrants who move along with their adult children generally face significant challenges to their mental wellbeing. Positive determinants of mental health are identified at the individual and family levels, and they include personal character, independent income, good physical health, social engagement, and familial economic and social support [4]. Meanwhile, negative determinants include perception of inferior economic status compared to local older people; intergenerational conflict; and institutional or policy disparities such as medical insurance, *hukou*, and pension institution [4].

A growing body of empirical research has shown that social capital generally has a positive effect in terms of promoting mental health and preventing mental illness. Putnam defined social capital as ‘connections among individuals—social networks and the norms of reciprocity and trustworthiness that arise from them’ [11]. Previous research has shown that the relationship between social capital and mental health is complex, and it is influenced by different dimensions and components, measurements, and contexts of the participants. Almedom [12] has advocated for a more detailed understanding of the various dimensions of social capital. This researcher described bonding social capital and bridging social capital as two distinct types of social capital, and cognitive social capital and structural social capital as two separate components or forms of social capital. Each type (i.e., bonding and bridging) has two components (i.e., cognitive and structural), operating at micro (individual or family/household) and/or macro (ecological, i.e., neighborhood, community, formal or informal group) levels. Structural bonding social capital refers to social networks, whereas cognitive bonding social capital refers to social control/efficacy, shared values, mutual trust, and norms of reciprocity. Structural bridging social capital refers to access to public goods, services, and amenities, whereas cognitive bridging social capital refers to participation, sense of belonging, and decision-making capacity [12]. 

**Figure 1:**
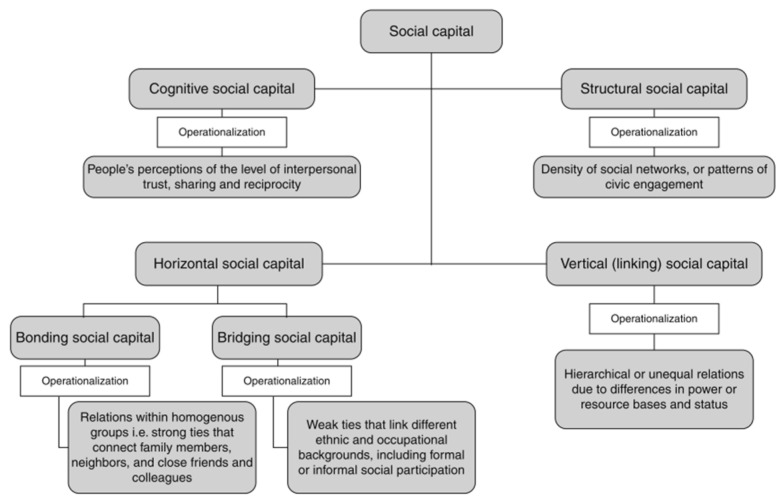
Dimensions of social capital and operationalization of the concept.

Villalonga-Olives and Kawachi [13] further proposed that social capital could be broken down and operationalized into two dimensions in the field of health research: a structural–cognitive dimension, and a bonding–bridging dimension (Figure 1).

*Structural social capital* refers to the externally observable behaviors and actions of actors within a network [13], and describes the properties of the networks, relationships, and institutions that bring people and groups together [14], with measures based on organizational membership, civic participation, and social interaction [15]. Structural social capital can provide people with access to institutions, both formal and informal, which may reduce the negative impacts of life events by providing additional support systems for older adults [16]. 

*Cognitive social capital* refers to people’s perceptions of levels of interpersonal trust, as well as norms of reciprocity within a group; it is often measured through an examination of individuals’ perceptions of trust and reciprocity [15]. Previous studies in different countries and regions have found that cognitive social capital is significantly related to mental health, with trust being identified as an independent predictor of individual health and being positively associated with health [16], as cognitive social capital may increase feelings of security and self-esteem and thus improve mental health [17]. 

Differently from the findings in Western countries, research on mental health and social capital (structural-cognitive dimension) among older people in China [18,19,20] has demonstrated that cognitive social capital (trust and reciprocity) is significantly associated with health outcomes (depression or QoL). Structural social capital (i.e., social participation, personal networks, neighborhood relationships and support) has no significant correlation with health outcomes, largely because formal organizations seldom exist in China [21,22]. 

*Bonding social capital*, according to Putnam [11], is by choice, a necessity, inward-looking, and tends to reinforce exclusive identities and homogeneous groups, while bridging (or inclusive) social capital is outward-looking and encompasses people across diverse social strata [11]. While bonding social capital involves strong ties and homogeneous networks, bridging social capital involves weak ties and heterogeneous networks [23]. Thus, bonding social capital intensifies existing support networks, and bridging social capital extends potential opportunities through the support network [23]. It echoes the notion of strong ties, such as ties with family and close friends, which provide a more intense, multi-stranded form of support and therefore may be expected to play a greater role in emotional wellbeing [23].

*Bonding social capital* is essential for mental health, especially in older people [24]. Family is among the most important mental health-promoting factors among older adults, and good and reliable friendships are perceived as an essential mental health resource, especially with people of a similar age who have more spare time for socializing than their family members of younger generations [23,24].

*Bridging social capital* echoes the notion of weaker ties, such as ties with acquaintances and various contacts, and can facilitate access to information and opportunities [24]. Empirical research has shown that bridging social capital is significantly associated with job opportunities and economic development for individuals and communities, including migrants [22]. However, the function of bridging social capital in terms of mental health has not been sufficiently researched [25,26], especially among aging people. Moreover, as Villalonga-Olives and Kawachi [15] pointed out, bridging and bonding social capital have been measured without specifying the heterogeneity or homogeneity of people involved in social network relationships. The changeable boundary between bonding and bridging capital causes difficulty in developing a common scale or questionnaire to measure these two dimensions of social capital. 

In order to more accurately measure bonding and bridging social capital, this research draws on frameworks from Almedom [12] and Villalonga-Olives [15], each of which will be classified into two components: cognitive and structural. Bonding social capital is classified into a bonding cognitive aspect (*Boc social capital)* and a bonding structural aspect (*Bos social capital)*, and bridging social capital is classified into a bridging cognitive aspect *(Brc social capital)* and a bridging structural aspect *(Brs social capital).* These are defined as follows.

*Boc social capital* (cognitive aspect of bonding social capital): this refers to perceptions of the level of interpersonal trust and reciprocity within homogenous groups, including family, close friends, and relatives, and connections with homogenous memberships (e.g., other OPMA).

*Bos social capital* (structural aspect of bonding social capital): this refers to social interaction within homogenous groups, including family, close friends and relatives, and connections with homogenous memberships (e.g., other OPMA).

*Brc social capital* (cognitive aspect of bridging social capital): this refers to perceptions of the level of interpersonal trust and reciprocity within heterogeneous groups, including neighborhoods and both formal and informal organizations in destination communities.

*Brs social capital* (structural aspect of bridging social capital): this refers to participation and membership within heterogeneous groups, including the neighborhood and both formal and informal organizations in destination communities.

Previous research has been concerned with the difficulties confronted by OPMA. These studies have highlighted the importance of considering the mental health of OPMA as a social issue, which requires structural changes at the community, societal, and policy levels. These studies also reflect a widening perspective on support for older people, beyond the context of ‘traditional’ Chinese cultural assumptions that elder care is a family responsibility [27]. 

However, on the whole, it seems that research on OPMA has only reached an initial stage. This is not only because of the limited quantity of published studies on OPMA (with no studies on OPMA published in top journals in mainland China during the review period), but also because previous studies lacked diversity in conceptualizations of mental health and in theoretical perspectives to enrich the understanding of the experiences of OPMA as well as potential interventions and supports [3]. 

Firstly, previous research on OPMA reflects a lack of clear definitions of mental health. For research studying mental health directly, adopting different indicators has caused different, and even conflicting, outcomes (e.g., depression vs. subjective happiness); for research focusing on adaptation or integration, much information was provided referring to mental health as a holistic status of OPMA, but mental health as a concept was rarely mentioned, resulting in mental health in terms of individual suffering being overlooked. 

Furthermore, few studies have examined the contributions of the factors influencing the mental health outcomes of OPMA. This area of research is crucial for guiding mental health practice through identifying the intervention focuses in this population. Hence, continuous research should delve into a more detailed analysis of the roles and interactions of these specific factors to address this research gap.

It has been suggested that both social cohesion and network definitions of social capital have merit in pointing to the existence of valued resources (capital) that are inherent within, and are by-products of, social relationships [26]. Thus, reflecting a pragmatic approach, this research will adopt Villalonga-Olives and Kawachi’s understanding of social capital and define social capital as resources embedded in networks, in a broad sense [13,15]. In particular, for the purposes of this research, social capital refers to networks and norms of trust and reciprocity within social relations. 

A limited number of studies have referred to the moderating or mediating effects on the relationship between social capital and mental health specifically for OPMA. Based on the findings of the literature review (described above), as well as related literature on social capital and mental health, some potential predicting factors that could be further examined are discussed below, including: gender, rural/urban background, living arrangement, and caregiving role.

Gender is a correlate that has frequently been mentioned in previous studies. Regarding OPMA, one study [28] reported that female OPMA reported a poorer quality of life in the physical, psychological, and environmental dimensions (WHOQOL-BREF), potentially related to their disadvantaged status and long time undergoing labor.

Urban/rural background can also affect the relationship between social capital and mental health, especially in China, where there are significant inequalities in terms of medical insurance and pension systems [29] and disparities in cultural and environmental settings between urban areas and rural areas. Previous research [19] has found that rural-to-urban OPMA report lower social capital and higher depression levels than their urban-to-urban counterparts. Rural–urban OPMA might experience a poorer capacity to adapt to urban life with new social norms, values, and customs, and to overcome cultural barriers such as different dialects, which may in turn reduce their ability to maintain and develop social capital [19].

Qualitative [3] and quantitative [30] studies have found that OPMA living with marital partners were more adaptive and happier than those without partners, due to greater feelings of safety and belonging, social engagement, and fewer negative emotions [30]. These findings suggest that, compared to OPMA who are not living with marital partners, those living with marital partners tend to have more bonding and bridging social capital as well as better mental health statuses.

Caregiving affects both mental health and social capital. On one hand, caregiving involves more labor-intensive activities and fewer enjoyable activities, and has been associated with a lower level of happiness among older Chinese adults [31]. Caregiving may also lead to more intergenerational conflict, which can contribute to greater emotional stress for OPMA [32]. On the other hand, caregiving means investing in family relationships, which can increase bonding social capital and can also provide more chances to interact and develop friendships with other OPMAs [33], which may help to build bonding social capital. 

To fill the research gaps and previous research findings mentioned above, the specific research questions in this study include: (1) What is the mental health status of OPMA? (2) What are the effects of bonding social capital and bridging social capital on mental health of OPMA?

## 2. Materials and Methods

### 2.1. Sampling

The targets of this study were OPMA in the city of Shenzhen, a well-known migration city in China. Data collection was carried out between November 2019 and January 2020. As there was no complete list of non-registered aging people migrating along with adult children in the community-level governmental offices, mixed quota sampling and purposive sampling strategies were used to identify the participants from selected districts and communities. According to data from a news report [30], there were approximately 960,000 older people living in Shenzhen without a local hukou in 2016. Using the Survey Monkey sample size calculator, setting the margin of error at 5%, the confidence level at 95%, and the power at >0.95, a sample size of 385 was determined. Thus, for this study, we planned to recruit a slightly larger sample of 400 OPMA participants.

A total of four districts, including two districts (Futian and Nanshan) from ‘Guannei’ (inner city, more urban-developed area) and two districts (Baoan and Longhua) from ‘Guanwai’ (outer city, less urban-developed area) were selected in Shenzhen. In each district, two communities per street were selected. The selection of the streets and associated communities was based upon the professional connections of the first author with these local administrative units. Then, from each community, 50 participants meeting the inclusion criteria were identified through personal networks. The included participants were aged 50 years and older, and had moved to live in Shenzhen from another urban/rural area for 6 months or more to join their adult children and to help to take care of grandchildren. The authors fully understand that people at the age of 50 years should not be considered older people. Given the fact that age 50 can be considered aging adults, and people at this age may have adult children and grandchildren, they were included if they met the other inclusion criteria of migrating to Shenzhen with or joining their adult children in order to take care of their grandchildren. The eligible participants were also cognitively and physically capable of communicating with the interviewers. The distribution of the sample in various districts and communities is presented in Table 1. A total of 399 valid cases were successfully included.

### 2.2. Data Collection

A structured questionnaire was administered through face-to-face interviews. The interviewers were five social workers, including the first author, working in community service centers for more than five years. They received interview training from the first author prior to data collection. Ethics approval for this study was received from the Human Subjects Ethics Sub-committee of the Hong Kong Polytechnic University (Reference number: HSEARS20190912001). The researcher provided the participants with a consent form explaining the purpose and nature of the study, and only proceeded with the research team after obtaining their informed consent. This ensured that participants’ privacy and confidentiality were protected throughout the study.

### 2.3. Measurement

To measure mental health, five variables were used.

*The 12-item General Health Questionnaire (GHQ-12) (Chinese version*). This consists of 12 items, each assessing the severity of a mental problem over the past few weeks, using a 4-point scale (from 0 to 3). The score was used to generate a total score ranging from 0 to 36, with higher scores indicating worse mental health conditions [34]. The GHQ-12 has been used with Chinese populations with good internal consistency and criterion validity [22,35]. 

*Self-perceived mental health.* This was measured by the question: Generally speaking, how do you rate your mental health status? The item was rated along a 5-point scale (1 = very bad, 5 = very good).

*Life satisfaction.* This was measured by a self-rated question: Generally speaking, how satisfied do you feel with your life? The item was rated along a 5-point scale (1 = very unsatisfied, 5 = very satisfied).

*Subjective happiness.* A single item, ‘How happy do you feel?’, was used to measure subjective happiness, with scores ranging from 1 (very unhappy) to 5 (very happy).

*Geriatric Depression Scale (GDS-15) (Chinese version).* GDS-15 has been widely used among the older Chinese population [29,36]. It comprises 15 items, each investigating the feelings and behaviors in the past one-week period. The participants were asked to rate each item with an answer of either yes or no. A score of 1 was assigned to an item indicating a depressive symptom. The total score ranged from 0 to 15, and the higher the score, the more depressive symptoms were present. When applied in a previous study, a Cronbach’s alpha of 0.873 was reported [37]. 

Four aspects of social capital were measured: the cognitive aspect of bonding social capital, the structural aspect of bonding social capital, the cognitive aspect of bridging social capital, and the structural aspect of bridging social capital. This study built upon and adapted the measurement used by Norstrand and Xu [21], which measured bonding and bridging social capital among older Chinese people. As Norstrand and Xu’s measurement items only included cognitive components (trust, closeness, and reciprocity) of the bonding and bridging social capital, this study added items for measuring structural components of both bonding and bridging social capital [21]. These structural component questions were adapted from previous social capital studies [19,38] which examined social contact, social participation, and memberships.

*Cognitive bonding social capital* (cognitive aspect of bonding social capital): To measure the cognitive components of bonding social capital (cognitive bonding social capital), questions were adapted from those used by Norstrand and Xu (reliability α = 0.706) [21]. These items included ten Likert questions on trust and closeness to the neighborhood, local older adults, other OPMA in their communities, fellow-townsmen, villagers *(lao xiang)*, classmates, colleagues, friends in their hometown, relatives, and family members. For each item, ‘most of them cannot be trusted’ was scored 1, ‘some of them cannot be trusted’ was scored 2, etc. and ‘most of them can be trusted’ was scored 5. The total scores on this scale ranged from 10 to 50, with higher scores indicating a higher level of cognitive bonding social capital.

*Structural bonding social capital* (structural aspect of bonding social capital): In order to measure the structural bonding social capital, a question was adapted from a study conducted by Li, Zhou, Ma, Jiang, and Li [19]: ‘On average, how many times a week do you and others (family members, friends, other OPMA) drop in on one another?’ and there were also ten items, neighborhood, local older adults, other OPMA in communities, fellow-townsmen/villagers (*lao xiang*), classmates, colleagues, friends at hometown, relatives and family members. ‘none’ was coded as 0, ‘1–2 times a week’ was coded as 1, ‘3–5 times a week’ was coded as 2, ‘more than 5 times a week’ was coded as 3. The total score for this scale ranged from 0 to 30, with higher scores indicating a higher level of structural bonding social capital.

*Cognitive bridging social capital* (cognitive aspect of bridging social capital): To measure the cognitive components of bridging social capital, questions were adapted from those used by Norstrand and Xu (reliability α = 0.704), including nine items on the frequency of reciprocity with organizational members [21]. Each item was rated along a five-point Likert scale, ranging from 0 (none) to 4 (most). The scores ranged from 0 to 36, and a higher score indicated a higher level of cognitive bridging social capital. 

*Structural bridging social capital* (structural aspect of bridging social capital): To measure the structural component of bridging social capital, a question was adapted from a study conducted by Lane, Wong, Mocnik, Song, and Yuen [38]: ‘In this community, have you participated in any type of groups or organizations in the past 12 months: (a) sports or recreational; (b) cultural, educational, or hobby; (c) religious-affiliated; (d) neighborhood, civic, or community; (e) the party members’ activities; and (f) others’ (multi-chosen items). The variables were coded according to the number of items, with scores ranging from 0 to 6. A higher score indicated more community memberships and a higher level of structural bridging social capital.

The Cronbach’s α values in the Boc, Bos, Brc, and Brs dimensions were 0.905, 0.0785, 0.896, and 0.836, respectively. Generally, when the Cronbach’s α value is greater than or equal to 0.7, it indicates that the scale has good reliability [35]. Therefore, the reliability of the four dimensions of the scales was good.

### 2.4. Confounding Variables

Demographic variables, living and caring arrangement variables, and physical health status were included as confounding variables, as many have been found to be related to mental health in the previous literature [39,40]. 

*Demographic variables* included gender, age, education, rural/urban background, retirement pension (‘have’ or ‘not’), perceived income adequacy (a 5-point Likert scale), residential length (number of the years living in Shenzhen, a continuous variable), expected residential length (no more than 6 years, 6 to 10 years, more than 10 years, or not sure), and marital status (married and living with partner, married and living apart from partner, or single). 

*Living and caring arrangement variables* included co-living adult children (whether one lives with son or daughter, both, or lives alone), living space (whether one shared a room with grandchild(ren) or resided in a single room or a house), frequency of returning to one’s hometown (the number of times one has returned to their hometown in the past 12 months, a continuous variable), age of the grandchild (age of the grandchildren that the participant is providing care for), housework time (amount of time spent per day on housework), and relationship with adult children. To measure the relationship with adult children, the Intergenerational Relationship Quality Scale (IRQS scale) (Chinese version) was used [35]. This scale has 13 items measured along a 5-point Likert scale, with the total scores ranging from 13 to 65. A higher score indicates a better relationship quality. 

*Physical health variables* included a self-rated question on one’s self-perceived physical health on the scale of 1 (very bad) to 5 (very good), and a binary yes or no question asking whether the participant had been diagnosed with an illness.

### 2.5. Data Analysis

Before data analysis, duplicated data were checked and cleaned, while missing data were handled using the mean replacement method. The study also employed histogram and Q-Q plot analyses to examine and confirm the normal distribution of the dependent variables before parametric statistics were used for further data analysis. Descriptive statistics were used to describe participants’ demographic characteristics, characteristics of their living and caring arrangements, and physical health characteristics. They also used bivariate statistics to determine how the characteristic variables related to each of the mental health measures.

The social capital was described within four components using descriptive statistics, and the bivariate relationship between characteristics variables and social capital within these four components was examined.

Finally, hierarchical multiple regression was used to test the relationship between the four social capital variables and each of the mental health variables, entering demographic variables, living and caring arrangement variables, physical health variables, and social capital variables into four successive blocks of predictors.

## 3. Results

The study aimed at examining the mental health status of OPMA and the effects of bonding social capital and bridging social capital on their mental wellbeing in China. In order to achieve this aim, we conducted a quantitative research survey among 399 OPMA participants. The descriptive results of the research are as follows. 

### 3.1. Demographic Background of the Participants

The participants in this study included 399 persons migrating along with their adult children. They were aged between 50 and 86, with an average age of 63.45 (SD = 6.69). Of the participants, 31.1 percent were men and 68.9 percent were women. Over half (56.6%) of the participants were urban–urban migrants who came from urban areas, and 173 participants (43.3%) were rural–urban migrants who came from rural areas. 

According to the urban–rural dual structural policies in China, residents in urban areas are enrolled in endowment insurance at their labor age via their work unit and receive a retirement pension every month after retirement [4]. However, farmers in rural areas cannot join the endowment insurance and have no retirement pension, unlike their urban counterparts. In this study, 193 participants received a retirement pension, and 206 participants had no retirement pension (Table 2).

### 3.2. Social Capital and Mental Health of the Participants

Table 3 presents the scores of the four types of social capital. The OPMA generally reported higher scores along the scoring range of the cognitive aspect of bonding social capital, while the lowest scores were found for the scoring range of the structural aspect of bridging social capital. 

Table 3 also shows the scores of the participants for the five mental health variables. The OPMA in this study mostly reported rather positive mental health statuses, with the mean GHQ and GDS scores falling in the lower end of the scoring range. At the same time, most of the participants reported positive choices in terms of self-reported mental health, life satisfaction, and happiness. However, it is also important to note that almost 25% of the OPMA reported a mild to moderate or severe level of depression. 

### 3.3. Relationships between Social Capital and Mental Health

Table 4 presents the regression analysis findings when each of the four mental health variables was used as the dependent variable. After controlling for variables related to demographics, living and caring arrangements, and physical health, some or most of the social capital variables were significantly associated with the mental health variables.

Among all the confounding variables, having a higher level of perceived income adequacy, a more positive relationship with adult children (except for GHQ score), and better self-reported physical health were found to be significant predictors of being more mentally well, meaning reporting lower GHQ and GDS scores and higher levels of happiness and life satisfaction.

Among the four social capital variables, the one related to the cognitive component of bonding social capital was not significant in its association with the four mental health variables. Those who reported higher levels of the structural component of bonding social capital also reported higher levels of happiness. A higher level of the cognitive component of bridging social capital was found to be associated with a greater degree of happiness and having a higher level of life satisfaction. Finally, those who reported a higher level of the structural component of bridging social capital also reported a lower level of GHQ and GDS scores, indicating fewer negative mental health and depressive symptoms. The standardized coefficients on the effects of the social capital variables ranged from 0.145 to 0.182, depending on the nature of the dependent variable.

## 4. Discussion

This research aimed at examining the mental health statuses of OPMA and the effects of bonding social capital and bridging social capital on their mental wellbeing in China.

In terms of the mental health of the OPMA, it is important to note that about one in five of them reported experiencing mild depression, while 4.3% reported moderate to severe levels of depressive symptoms [19]. These findings are consistent with previous research on older Chinese adults, which found that 31.2% of older migrants reported mild to severe depression as measured by GDS-30 in Hangzhou [19].

Among the various social capital variables, participants reported higher levels of bonding social capital than bridging social capital. For most of them, family members were their main or only social ties and sources of support [41,42,43,44]. The main social interactions for most of these older people took place in the neighborhood by chance and were unplanned. Therefore, these findings are close to the reality faced by older adults moving along.

However, the structural component score of social capital was 1.28, meaning that each older migrant had, on average, participated in more than one group or organization. This level is likely to be higher than the level of older people migrating along in other cities. For instance, in other studies, most participants reported no social activities during their leisure time in the cities of Jiangsu province [45,46,47]. This difference in result may be due to three reasons. Firstly, Shenzhen is a migration city where most residents are migrants moving from other cities, and the older people who were born in Shenzhen are rare [20]. Therefore, many OPMA may not see themselves as outsiders when they interact with other older people who are mostly migrants from other places. This unique social context facilitates their social engagement with others. On the other hand, the so-called local Shenzhenese tend to see themselves as migrants as well, probably facilitating their welcoming attitude to other older people who move to Shenzhen with their adult children. Secondly, the local Shenzhen government provides some community services. In recent years, the local government has built a community service center in each community in Shenzhen, where different kinds of activities are hosted and interest classes and groups have been developed to enrich residents’ social lives [20]. All these services are supported by the government and are free for all residents, including those without a registered ‘hukou’ (household registration) [4]. The ease of accessibility to these community services for older migrants may be a factor in their easy engagement with other residents in the community. Thirdly, there are many free provisions under the parity policy in Shenzhen. For instance, all older people, with or without ‘hukou’, can enjoy the use of free public transport and free entry into public parks and recreational facilities, thereby facilitating their social activities and enhancing social engagement [4,20]. These measures appear to be successful in terms of enhancing the structural social capital of older people, particularly that of older migrants.

Our major distinctive finding involves the role of bridging social capital in mental health. The finding is very different from, and is even in contradiction with, previous research conducted in China [21,22]. Previous research [21] found that bridging social capital (measured as the extent to which people help each other or expect to be helped in organizations in which they have participated) was not significant for either urban or rural older adults in China. However, this study finds that bridging social capital plays a significant role in terms of mental health, with four indicators: life satisfaction, subjective happiness, GDS, and GHQ. These findings provide adequate evidence that maintaining social interactions and joining or being affiliated with groups and organizations in the community are more important than generating a subjective sense of trust and reciprocity within their social circles.

This is likely due to three reasons. Firstly, Shenzhen provides plenty of public services and open policies for joining community organizations supported by the local government, such as senior chorus and Tai Chi classes. This means that OPMA in Shenzhen should have relatively easy access to more public services and community organizations than OPMA in other cities, and this can be beneficial to the building of bridging social capital. Secondly, Shenzhen is a migrant city, and most older people are migrants. This is very different from other cities in China, where local older adults are the main component of the older population. OPMA in Shenzhen could more comfortable and positive in terms of making friends with other older people, who are also mostly migrants. Thus, their sense of social inferiority and social exclusion might be less intense when interacting with other older people who share similar backgrounds as migrants. Finally, the difference may be due to the unique development of community and social organizations in Shenzhen [20]. In the past, when social organizations were not well developed, older people had social interactions mainly with strong social ties (bonding social capital). However, currently, in Shenzhen, older people (including OPMA) have begun to extend their social interactions to ‘strangers’ and community service organizations.

Another distinctive finding of this study is that cognitive bonding social capital was found to have no relationship with any mental health variables. This finding is different from previous research on older people, which indicates that social capital has positive effects on mental health for older people through their closest family members, who can provide support and feelings of being loved, and good friends of similar age [24]. This finding could be due to two reasons. Firstly, it could be related to measurement. The bonding social capital in this study included social ties not only with family, relatives, and close friends, but also with neighbors. Thus, the function of perceived trust and reciprocity from family, relatives, and close friends may have been affected by the inclusion of measuring the ties with neighbors. By further checking the relationship between the IRQS variable (relationship with adult children) and mental health in this study, it becomes noticeable that IRQS has a significant impact on depression, life satisfaction, and subjective happiness, which is consistent with findings from previous research [24]. A second consideration is the breaking of social ties with close friends for OPMA. As OPMA have left their close friends and relatives in their hometowns, their social ties and perceptions of trust and reciprocity may have weakened as time has passed. They may, therefore, struggle to obtain significant support from close friends and relatives in their hometowns. This is very different from local older residents who have grown old in their home communities and receive strong support from the close friends nearby. 

The third distinctive finding concerns the function of social interaction with local older people. Previous research [25] has shown that interaction with local older people brings about a perception of social inferiority and social exclusion for older people who move along. This would further generate a negative impact on the older migrants’ mental health. However, our research has found that this is not the case in Shenzhen. A probable reason may be that in Shenzhen, the distinctions between ‘local’ and ‘migrant’ are blurry since Shenzhen is a city made up of mainly migrants, making the concept of local or native Shenzhenese a misnomer. Moreover, both OPMA and ‘local older people’ speak Mandarin rather than a local Shenzhen dialect, which does not exist. Therefore, there is no obvious distinction in language identity, and no obvious difference with which to form a basis of social exclusion.

### 4.1. Implications for Practice

All of our findings have shown that building bridging social capital is key to improving mental health for OPMA. Bridging social capital has a significant effect on four mental health indicators, namely, GHQ, GDS, subjective happiness, and life satisfaction. It also means that enhancing bridging social capital can promote mental health in multiple aspects, both improving positive mental health and preventing mental illness.

Social capital is ‘growable’, as it is positively related to age, length of residence, and social connections or interactions. This means that if OPMA reside in Shenzhen for a longer period of time, they have the potential to enhance their bridging social capital. To achieve this, services in the communities should be offered to aid older people in gaining more opportunities to join different groups and organizations via social activities and events. Practitioners working with these older migrants should be familiar with different types of activities and ways of connecting OPMA with these activities or organizations.

Practitioners working with older people should play an important role in building social capital in the community, particularly for older people who have moved along with their adult children during the first few years of settlement. Based on the finding that bridging social capital is beneficial for OPMAs’ positive mental health, an effective method of building bridging social capital is to facilitate them to participate in activities or join organizations. Practitioners, as brokers of services, could connect these older people to mass organizations. They should gain adequate understanding of the nature of the services and make professional judgements on how different activities may benefit the unique social capital and mental health needs of older people. 

Secondly, the government and service providers should further enhance older people’s and their adult children’s understanding of social participation. Relevant measures to provide information about community events and activities via relevant digital platforms for older migrants are important. Yet another challenge is to educate adult children who may be overprotective at times and who are unwilling to have their aging parents spend too much time out of the home due to perceived risks of accidents, scams, or health concerns. These overprotective attitudes may discourage older people from building their own social networks and social capital. Practitioners can play an advocate role by working with adult children and their family members and sensitizing them to the social capital and mental health needs of OPMA.

Thirdly, promoting knowledge about mental health and mental illness is important for older migrants and their families. Strong support from adult children is likely to enhance OPMAs’ subjective happiness and facilitate them to build bridging social capital and actively integrate themselves into the new environment, thereby improving their mental health.

Fourthly, certain family social work should be provided to ‘high risk’ families. This study has indicated that a poor relationship with adult children is associated with more depressive symptoms among OPMA. Depression further hinders OPMA from building bonding social capital and bridging social capital. In this case, professional family social workers should have a key role in mending the relationship between adult children and OPMA.

Finally, social capital assessment and enhancement could be the practice framework for practitioners to work with clients who are displaced, marginalized, or lacking social connections and affiliations. By assessing the social capital status of each client, particularly that of older migrants and others who have moved along with their families, their needs can be further identified. 

### 4.2. Implications for Future Research

This study addressed social capital by examining its bridging–bonding and structural–cognitive dimensions. This is a response to the suggestions made by other researchers [15] who consider that social capital covers different constructs and that it is necessary to specify the specific components of social capital. The specific measurements of social capital which we used cover a wider range of social capital components, facilitating the identification of the specific effects of different dimensions of social capital on mental health.

While previous research in China has generally indicated that bridging social capital has no significant effects on older people’s health [47], this study has shown otherwise. It is likely that for migrant older people who have uprooted themselves from their hometown, with reduced social connections and affiliations, bridging social capital that helps them to reconnect with others is crucial. Further research should be conducted to differentiate the effectiveness of different types of groups, organizations, affiliations, events, and activities in enhancing one’s bridging social capital. Although bonding social capital is not as significant in predicting mental health, the extent to which the enhancement of bridging social capital may facilitate the enhancement of bonding social capital should also be examined. 

### 4.3. Limitations

Three main limitations must be noted. Firstly, this study used a non-probability sampling with a small sample size of only 399 participants. Therefore, the findings carry limited generalization power to all OPMA in Shenzhen. Given the study’s focus on one city, the findings also cannot represent the situations of OPMA in other cities in China. Second, as a cross-sectional study using regression analysis to examine the relationship between social capital and mental health, the causal relationship between social capital and mental health cannot be established. The third limitation of this study is the absence of pre-data-collection validity assessments for all scales, which could potentially impact the credibility and generalizability of the study’s results.

## 5. Conclusions

The economic development in China in recent decades has helped young adults to gain additional employment and career opportunities away from their hometowns or communities. With their enhanced standard of living, more people have the financial capacity to bring their aging parents or grandparents to join them. In this process, OPMA contribute by performing household chores and caring for their grandchildren unpaid. Meanwhile, they experience challenges and needs associated with social and mental well-being. While bonding social capital correlates with only one indicator of mental health, bridging social capital is significantly related to other mental health indicators. Through strengthening bridging social capital, these older adults can benefit from more opportunities for participation in formal or informal organizations in their communities and improve their mental wellbeing.

## Figures and Tables

**Table 1:** Sampling distribution.

District	Street	Community	Participants
Futian	Xiang Mi Hu	Nong Yuan	50
(more urban-developed area)		Dong Hai	50
Nanshan	Zhao Shan	Hua Guo Shan	50
(more urban-developed area)		Shui Wan	50
Bao’an	Xi Xiang	Fu Zhong Fu	50
(less urban-developed area)		Tang Wei	50
Longhua	Da Lang	Shang Ling Pai	50
(less urban-developed area)		Luo Wu Wei	50

**Table 2:** Demographic characteristics of participants (n = 399).

Sample Characteristics	N (%) or M (SD)
Gender	
Male	124 (31.1)
Female	275 (68.9)
Age	63.45 (6.69)
Education	
Primary school or below	147 (36.8)
Junior high school	104 (26.1)
Senior high school	93 (23.3)
College degree or above	55 (13.8)
Hometown	
Urban areas	226 (56.6)
Rural areas	173 (43.4)
Marital status	
Married and living with spouse	224 (56.1)
Married and living apart from spouse	112 (28.1)
Single	63 (15.8)
Pensions	
Have pension	193 (48.4)
No pension	206 (51.6)
Finance resource (multiple choice)	
Pension	190 (47.6)
Alimony	201 (50.4)
Labor income	40 (10.0)
Others (investment, saving etc.)	48 (12.0)
Income adequacy (1–5)	3.31 (0.79)
Length of residency in Shenzhen	7.08 (5.36)
Expected length in Shenzhen	
No more than six years	39 (9.8)
Six to ten years	45 (11.3)
More than ten years	115 (28.8)
No plan and not sure	200 (50.1)

**Table 3:** Descriptive results of social capital and mental health variables.

Social Capital Variables	Score Range	n (%)	M (SD)
Cognitive bonding social capital	10–50	-	39.46 (7.08)
Structural bonding social capital	0–30	-	12.73 (5.70)
Cognitive bridging social capital	0–36	-	15.18 (7.15)
Structural bridging social capital	0–6	-	1.28 (1.19)
**Mental health variables**			M (SD)
GHQ (range 0–36)			5.71 (4.80)
Self-report mental health	1–5	-	3.69 (0.75)
Life satisfaction	1–5	-	3.74 (0.78)
Subjective happiness	1–5	-	3.72 (0.77)
GDS	0–15	-	3.39 (2.58)
Normal	0–4	301 (75.4)	
Mild depression	5–9	81 (20.3)	
Moderate to severe depression	10–15	17 (4.3)	

**Table 4:** Regression analysis of social capital and mental health variables.

		GHQ	GHQ-SE	*p*-Value	Happiness	Happiness-SE	*p*-Value	Life sat.	Life-sat-SE	*p*-Value	GDS	GDS-SE	*p*-Value
		Beta			Beta			Beta			Beta		
Demographics													
	Age	0.080	0.038	10.530	−0.004	0.007	0.942	0.029	0.007	0.629	−0.023	0.021	0.675
	Gender (ref: male)	−0.098 *	0.485	0.037	−0.079	0.087	0.131	0.000	0.090	0.998	−0.075	0.277	0.132
	Education	0.013	0.250	0.809	−0.022	0.045	0.726	−0.058	0.048	0.364	0.073	0.143	0.216
	Hometown (ref: rural)	0.015	0.527	0.782	0.097	0.094	0.111	−0.062	0.098	0.324	0.031	0.301	0.589
	Pension (ref: having a pension)	−0.087	0.616	0.176	0.099	0.110	0.167	−0.020	0.114	0.786	0.000	0.352	0.995
	Income adequacy (1–5)	−0.100 *	0.273	0.026	0.128 *	0.049	0.010	0.176 **	0.051	0.001	−0.160 **	0.156	0.001
	Marital status (ref: married and living together)												
	Married and living apart	−0.045	0.505	0.343	0.061	0.090	0.246	0.048	0.094	0.380	−0.038	0.288	0.455
	Single	−0.002	0.603	0.972	−0.042	0.108	0.406	−0.033	0.112	0.532	0.063	0.344	0.195
	Residential length	0.092	0.045	0.068	−0.033	0.008	0.556	−0.046	0.008	0.428	0.027	0.026	0.615
	Expected length in Shenzhen (ref: >10 years)												
	<6 years	0.056	0.771	0.241	0.004	0.138	0.940	0.047	0.143	0.386	0.100 *	0.440	0.050
	6–10 years	0.098 *	0.712	0.038	0.048	0.127	0.353	0.006	0.132	0.914	0.056	0.406	0.261
	Not sure	0.126 *	0.526	0.022	−0.048	0.094	0.431	−0.024	0.098	0.705	0.048	0.300	0.412
Living and caring arrangements													
	Co-living adult children (ref: with son)												
	with daughter	−0.119 **	0.478	0.008	−0.031	0.085	0.529	0.069	0.089	0.177	−0.035	0.273	0.465
	with both son and daughter	−0.039	0.919	0.350	−0.014	0.164	0.764	0.022	0.170	0.639	0.015	0.535	0.743
	with no son or daughter	−0.083	0.887	0.059	0.030	0.159	0.545	0.088	0.165	0.081	−0.067	0.506	0.151
	Frequency of returning to hometown	−0.067	0.157	0.115	−0.066	0.028	0.161	−0.014	0.029	0.781	−0.009	0.089	0.838
	Age of the cared-for grandchild	0.004	0.059	0.932	0.000	0.010	0.998	−0.016	0.011	0.747	−0.080	0.033	0.091
	Housework time	0.089	0.287	0.054	−0.032	0.051	0.536	−0.007	0.053	0.901	0.140 **	0.164	0.004
	IRQS (relationship with adult child)	−0.056	0.040	0.235	0.218 **	0.007	0.000	0.231 **	0.007	0.000	−0.171 **	0.023	0.001
	Living space—sharing a room with grandchild	−0.002	0.425	0.961	−0.003	0.076	0.967	−0.011	0.079	0.818	−0.010	0.242	0.835
Physical health													
	Self-reported physical health (1–5)	−0.360 **	0.311	0.000	0.180 **	0.056	0.001	0.165 **	0.058	0.004	−0.243 **	0.178	0.000
	Having an illness	0.112 *	0.450	0.017	0.046	0.080	0.379	0.033	0.084	0.535	0.062	0.257	0.213
Social capital													
	Cognitive bonding social capital	−0.045	0.030	0.306	0.003	0.005	0.944	0.025	0.006	0.628	−0.088	0.017	0.061
	Structural bonding social capital	−0.034	0.040	0.477	0.182 **	0.007	0.001	0.079	0.007	0.144	−0.063	0.023	0.207
	Cognitive bridging social capital	0.000	0.035	0.994	0.156 **	0.008	0.007	0.145 *	0.006	0.015	0.038	0.020	0.484
	Structural bridging social capital	−0.155 **	0.213	0.004	−0.066	0.038	0.259	−0.102	0.040	0.092	−0.166 **	0.122	0.003
	F(398)	90.543			50.118			30.951			60.859		
	Total R^2^	0.400			0.263			0.216			0.324		
	Adjusted R^2^	0.358			0.212			0.162			0.277		

Note: * *p* < 0.05, ** *p* < 0.01.

## Data Availability

The data presented in this study are available on request from the first author. The data are not publicly available due to data ownership and privacy reasons.

## References

[1] National Health and Family Planning Commission of the People’s Republic of China (NHFPC) (2016). Zhongguo Liu Dong Ren Kou Fa Zhan Bao Gao [Report on China’s Migrant Population Development]. http://www.nhc.gov.cn/rkjcyjtfzs/pgzdt/201610/57cf8a2bbafe4b4d9a7be10d10ae5ecf.shtml.

[2] Central Committee of the Communist Party of China, & State Council (2014). National New-Type Urbanization Plan (2014–2020). http://english.www.gov.cn/policies/policy_watch/2014/08/23/content_281474983027472.htm.

[3] Wang J., Lai D.W.L. (2022). Mental health of older migrants migrating along with adult children in China: A systematic review. Ageing Soc..

[4] Liu J. (2016). Ageing in rural China: Migration and care circulation. J. Chin. Sociol..

[5] Wang Y., Wang L., Wu H., Zhu Y., Shi X. (2019). Targeted poverty reduction under new structure. China Agric. Econ. Rev..

[6] Gu L., Cheng Y., Phillips D.R., Rosenberg M., Yang L., Wang L., Li H. (2021). Does social capital interact with economic hardships in influencing older adults’ health? A study from China. Int. J. Equity Health.

[7] Cui J., Lancaster K., Newman C.E. (2019). Making the subjects of mental health care: A cross-cultural comparison of mental health policy in Hong Kong, China and New South Wales, Australia. Sociol. Health Illn..

[8] Xue X., Reed W.R., Menclova A. (2020). Social capital and health: A meta-analysis. J. Health Econ..

[9] Dodge R., Daly A., Huyton J., Sanders L. (2012). The challenge of defining wellbeing. Int. J. Wellbeing.

[10] Stenius K. (2007). Promoting Mental Health. Concepts, emerging evidence, practice. Addiction.

[11] Putnam R.D. (2000). Bowling Alone: The Collapse and Revival of American Community.

[12] Almedom A.M. (2005). Social capital and mental health: An interdisciplinary review of primary evidence. Soc. Sci. Med..

[13] Villalonga-Olives E., Kawachi I. (2015). The measurement of bridging social capital in population health research. Health Place.

[14] Alvarez E.C., Romani J.R. (2017). Measuring social capital: Further insights. Gac. Sanit..

[15] Villalonga-Olives E., Kawachi I. (2015). The measurement of social capital. Gac. Sanit..

[16] Giordano G.N., Bjork J., Lindstrom M. (2012). Social capital and self-rated health: A study of temporal (causal) relationships. Soc. Sci. Med..

[17] Harpham T., Grant E., Rodriguez C. (2004). Mental health and social capital in Cali, Colombia. Soc. Sci. Med..

[18] Shen Y., Yeatts D.E., Cai T., Yang P.Q., Cready C.M. (2014). Social Capital and Self-Rated Health Among Middle-Aged and Older Adults in China: A Multilevel Analysis. Res. Aging.

[19] Li Q.J., Zhou X.D., Ma S., Jiang M.M., Li L. (2017). The effect of migration on social capital and depression among older adults in China. Soc. Psychiatry Psychiatr. Epidemiol..

[20] Zhong B.L., Liu T.B., Chan S., Jin D., Hu C.Y., Dai J., Chiu H. (2018). Common mental health problems in rural-to-urban migrant workers in Shenzhen, China: Prevalence and risk factors. Epidemiol. Psychiatr. Sci..

[21] Norstrand J.A., Xu Q. (2012). Social capital and health outcomes among older adults in China: The urban-rural dimension. Gerontologist.

[22] Yip W., Subramaniana S.V., Mitchella A.D., Leeb D.T.S., Wang J., Kawachia I. (2007). Does social capital enhance health and well-being? Evidence from rural China. Soc. Sci. Med..

[23] Halpern D. (2005). Social Capital.

[24] Forsman A.K., Herberts C., Nyqvist F., Wahlbeck K., Schierenbeck I. (2013). Understanding the role of social capital for mental wellbeing among older adults. Ageing Soc..

[25] Zhong Y., Schon P., Burstrom B., Burstrom K. (2017). Association between social capital and health-related quality of life among left behind and not left behind older people in rural China. BMC Geriatrics.

[26] Kawachi I., Subramanian S.V., Kim D. (2008). Social Capital and Health.

[27] Ngan R., Kwan A. (2002). The mental health status and long-term care needs of the Chinese elderly in Hong Kong. Soc. Work Health Care.

[28] Hu Y.X., Long L.L., Yin Y.Q. (2013). Cheng shi lao piao zu de sheng ming zhi liang ji qi ying xiang yin su fen xi [Investigation on the quality of life and its influencing factors for the elderly migrants in city]. Zhongguo Xian Dai Yi Sheng.

[29] Zhao Q.R., Brosig S., Luo R.F., Zhang L.X., Yue A., Rozelle S. (2016). The new rural social pension program in rural China: Participation and its correlates. China Agric. Econ. Rev..

[30] Chen S.G. (2015). Sui qian lao ren cheng shi shi ying ying xiang yin su de shi zheng yan jiu [Empirical study on the factors affecting the urban adaptation of the elderly migrating to city]. Fujian Nong Lin Da Xue Xue Bao (Zhe Xue She Hui Ke Xue Ban).

[31] Xu H., Mou S., Xu J., Zeng M.Y. (2014). Beijing di qu sui qian zhong lao nian ren de zhu guan xing fu gan ji qi xiang guan yin su [Subjective well-being and related factors for older people migrating along in Beijing]. Zhongguo Lao Nian Xue Za Zhi.

[32] He H.T. (2014). Dai ji guan xi shi jiao xia ‘lao piao zu’ de cheng shi shi ying yan jiu [Social adaptation for older drifting group: On perspective of intergeneration]. Qian Yan.

[33] Bao F.C. (2017). Sui qian lao ren de cheng shi shi ying wen ti tan xi [A deep exploration to the urban adaptation of the trailing eld]. Lanzhou Gong Ye Xue Yuan Xue Bao.

[34] McDowell I., Newell C. (1996). Measuring Health: A Guide to Rating Scales and Questionnaires.

[35] Qin M., Vlachantoni A., Evandrou M., Falkingham J. (2018). General Health Questionnaire-12 reliability, factor structure, and external validity among older adults in India. Indian J. Psychiatry.

[36] Wei J., Zhang J., Deng Y., Sun L., Guo P. (2018). Suicidal ideation among the Chinese elderly and its correlates: A comparison between the rural and urban populations. Int. J. Environ. Res. Public Health.

[37] Zhao H., He J., Yi J., Yao S. (2019). Factor Structure and Measurement Invariance Across Gender Groups of the 15-Item Geriatric Depression Scale Among Chinese Elders. Front. Psychol..

[38] Lane A.P., Wong C.H., Mocnik S., Song S., Yuen B. (2019). Association of neighborhood social capital with quality of life among older people in Singapore. J. Aging Health.

[39] Wright L.Q. (2019). The Predictability of Selected Demographic Factors on the Mental Health and Mental Health Disorders Among African American Adolescents. Ph.D. Thesis.

[40] Hanuscin C., Zahmatkesh G., Shirazi A., Pan D., Teklehaimanot S., Bazargan-Hejazi S. (2018). Socio-demographic and mental health profile of admitted cases of self-inflicted harm in the US population. Int. J. Environ. Res. Public Health.

[41] Bai X. (2018). Development and Validation of a Multidimensional Intergenerational Relationship Quality Scale for aging Chinese parents. Gerontologist.

[42] Lu N., Liu J., Lou V.W. (2015). Caring for frail elders with musculoskeletal conditions and family caregivers’ subjective well-being: The role of multidimensional caregiver burden. Arch. Gerontol. Geriatr..

[43] Ruan Y., Zhou L. (2022). Reactions of Chinese “drifting elderly” aged 50 years and over when facing negative experiences in urban living. Soc. Behav. Personal..

[44] Silverstein M., Cong Z., Li S. (2006). Intergenerational transfers and living arrangements of older people in rural China: Consequences for psychological well-being. J. Gerontol. Ser. B Psychol. Sci. Soc. Sci..

[45] Wu Z.J., Song Y., Wang H.L., Zhang F., Li F.H., Wang Z.Y. (2019). Influence of the built environment of Nanjing’s Urban Community on the leisure physical activity of the elderly: An empirical study. BMC Public Health.

[46] Cheng Y., Rosenberg M., Winterton R., Blackberry I., Gao S. (2019). Mobilities of older Chinese rural-urban migrants: A case study in Beijing. Int. J. Environ. Res. Public Health.

[47] Zhang Y., Jiang J. (2018). Social Capital and Health in China: Evidence from the Chinese General Social Survey 2010. Soc. Indic. Res..

